# Effect of nanoparticle incorporation on antimicrobial activity and shear bond strength of orthodontic adhesives: a scoping review

**DOI:** 10.1007/s44445-025-00038-7

**Published:** 2025-10-15

**Authors:** Nathalie Murielly Rolim de Abreu, Frederico Barbosa de Sousa, Rudyard dos Santos Oliveira

**Affiliations:** 1 Postgraduate Program in Dentistry, Federal University of Paraíba, Cidade Universitária S/N - Campus 1 Castelo Branco, João Pessoa, PB 58051-900 Brazil; 2 Morphology Department, Federal University of Paraíba, Conj. Pres. Castelo Branco III, João Pessoa, PB 58051-900 Brazil; 3 Postgraduate Program in Dentistry, Federal University of Paraíba, Rua Cantora Maria da Glória Gouveia de Vasconcelos, João Pessoa, PB, 58037-313 Brazil

**Keywords:** Nanoparticle-modified adhesives, Orthodontic bonding, Enamel demineralization, Clinical translation

## Abstract

Nanoparticle incorporation into orthodontic adhesives has emerged as a strategy to enhance antimicrobial properties, improve mechanical performance, and prevent enamel demineralization during fixed appliance therapy. This scoping review aimed to synthesize evidence on the effectiveness of nanoparticle-modified orthodontic adhesives. A systematic search and independent screening process were conducted by two reviewers across multiple databases following the PRISMA-ScR guidelines. Among the 153 initially identified records, 25 studies met the inclusion criteria. Most studies (72%) were in vitro, whereas only 12% used in situ models. Preclinical studies are scarce (4%), emphasizing the gap between laboratory findings and clinical application. Silver nanoparticles (AgNPs), which demonstrate strong antimicrobial activity against *S. mutans*, *E. coli*, and *S. aureus* while maintaining acceptable bond strength at moderate concentrations, have been the most frequently investigated. Other nanoparticles, such as titanium dioxide (TiO₂), β-AgVO₃, mesoporous bioactive glass nanoparticles (MBNs), calcium phosphate compounds, and calcium phosphate compounds, also show promising antimicrobial and remineralization properties. However, variations in study designs, nanoparticle concentrations, and adhesive formulations hinder direct comparisons. Despite promising laboratory findings, the clinical application of nanoparticle-modified orthodontic adhesives remains uncertain due to the lack of standardized methodologies and long-term clinical validation. Further well-designed clinical trials are essential to confirm their efficacy, safety, and impact on orthodontic bonding performance under real-world conditions. Standardization of nanoparticle formulations and biocompatibility assessments are crucial to ensure their practical integration into orthodontic practice, promoting safer and more effective treatments.

## Introduction

Fixed orthodontic appliances are indispensable tools in corrective orthodontic treatment. However, their use is associated with significant clinical challenges, such as the formation of carious enamel lesions around the brackets and bonding failures. These problems are largely due to the difficulty of sanitizing the areas adjacent to the brackets, which favor the accumulation of bacterial biofilms and, consequently, caries lesion formation. As a result, patients can face high treatment costs, compromised aesthetic results, and longer treatment times, reinforcing the need for innovative solutions in orthodontic practice (Yi et al. 2019; Nam et al. 2019; Farzanegan et al. 2021).

Faced with these challenges, researchers have explored different strategies to improve orthodontic adhesives by combining antimicrobial properties with adequate mechanical strength. One of the most promising approaches involves the incorporation of nanoparticles (NPs) into resin composites, an innovation that has the potential to revolutionize clinical orthodontics. Recent studies have shown that materials containing NPs can inhibit the formation of bacterial biofilms and simultaneously maintain the mechanical properties of adhesives, depending on the concentration of nanoparticles added (Mohammad et al. 2022; Muhammad et al. 2022).

Various nanoparticles have been investigated for their antimicrobial and mechanical potential. Silver nitrate (AgNP), widely known for its antimicrobial properties, has shown efficacy in its pure form and when combined with augmentin (Muhammad et al. 2022; Ahmed et al. 2022). An alternative that is gaining prominence is the use of titanium dioxide (TiO_2_), which is known for its antimicrobial properties and biocompatibility, although some studies indicate that its incorporation may reduce the shear strength (SBS) of composites (Poosti et al. 2013; Kotta et al. 2020; Mollabashi et al. 2023; Tivanani et al. 2024). Similarly, graphene nanoparticles combined with silver and vanadate with silver nanoparticles (βAgVO_3_) have shown promising results in bacterial inhibition (Nozha et al. 2022; Uehara et al. 2024).

Bioactive materials also occupy a prominent place in dentistry. Mesoporous bioglass (MBN) and its combinations, such as silver-associated bioglass (nBG@A) or 2-methacryloyloxyethyl phosphorylcholine monomer (MPC), have been widely explored because of their ability to remineralize enamel and improve biocompatibility (Park et al. 2020; Choi et al. 2021; Seifi et al. 2024). In addition, inorganic nanoparticles, such as amorphous calcium phosphate (ACP), n-hydroxyapatite, and arginine-loaded mesoporous silicate (Arg@MsNs), have shown the potential to prevent and remineralize enamel demineralization (Zhang et al. 2016; Xie et al. 2017; Melo et al. 2017; Ma et al. 2017; Dunn 2007; Rahmanpanah et al. 2023; An et al. 2024). Other innovative formulations include the combination of calcium phosphate monohydrate with strontium-containing bioactive glass nanoparticles (Sr/CaP) and andrographolide, as well as hybrid materials such as polycaprolactone-gelatin-silver nanoparticles (PCL-gelatin-AgNPs). These systems have robust antimicrobial properties and stand out for their versatility in clinical application (Chaichana et al. 2022; Yuan et al. 2022).

Nanoparticles derived from natural compounds, such as emodin (ENPs), have also received attention. Recent studies have indicated that adequate concentrations of ENPs can improve antimicrobial activity without compromising SBS. In addition, the combination of ENPs with techniques such as antimicrobial photodynamic therapy (aPDT) has demonstrated efficacy in reducing bacterial biofilm formation (Mirhashemi et al. 2024).

Although significant advances have been made, the results concerning the impact of different nanoparticles on orthodontic adhesives have been mixed. While some formulations demonstrate excellent antimicrobial activity with minimal effects on shear bond strength, others present limitations that compromise their practical application depending on the ideal insertion concentration of the nanoparticles.

This study seeks to address these gaps by systematically reviewing the literature concerning the incorporation of nanoparticles into orthodontic adhesives. The aim of this study was to evaluate the impact of these nanoparticles on both antimicrobial properties and shear bond strength, contributing to the development of more efficient and safer materials. The null hypothesis most commonly postulated in the included studies is that the addition of different nanoparticles does not negatively affect the mechanical strength of adhesives while optimizing their antimicrobial properties, thus contributing to the establishment of new parameters for evidence-based orthodontic practice.

## Materials and methods

This study is a scoping review that consists of synthesizing research evidence to map the literature on a given subject in terms of nature, characteristics, and volume (Arksey & O'Malley 2005). The review was conducted following the Joanna Briggs Institute methodology for scoping reviews (The JoAnna Briggs Institute, (2014)) and is in line with the PRISMA-ScR guidelines (Preferred Reporting Items for Systematic Reviews and Meta-Analyses Extension for Scoping Reviews) (Pollock et al. 2022). The research protocol was made publicly available on the Open Science Framework (doi.org/10.17605/OSF. IO/THVFS), according to methodological recommendations (Foster & Deardorff 2017; Poirier 2023).

To guide the formulation of this material, the following objective was adopted: Will orthodontic adhesives doped with nanoparticles present superior shear bond strength, prevention of enamel demineralization and antimicrobial activity than traditional adhesives without nanoparticles in natural human teeth?

A preliminary search of the PubMed, Scopus, and research protocol databases indicated that no corresponding review had been published or was in progress.

### Eligibility criteria

Primary and secondary quantitative or qualitative studies were included in the review. Theses, dissertations, books, and technical and government documents were also considered, with no time limit for selection. Publications in any language containing the following descriptors or keywords were included: dental bonding, curing of orthodontic adhesives, adhesives, nanoparticle-modified orthodontic adhesives, nanoparticles, nanocrystalline materials, nanocrystals, orthodontic brackets, dental braces, orthodontic braces, orthodontic friction, dental enamel, enamel, enamel cuticle, demineralization of tooth enamel, tooth demineralization, and shear bond strength.

The exclusion criteria were those that did not have as their main objective the evaluation of orthodontic adhesives with nanoparticles; studies using bovine, porcine, equine, or other teeth; and studies that did not evaluate shear bond strength or demineralization or antimicrobial activity.

### Information sources and search strategy

The search for scientific production was carried out in the following databases: PubMed, Scopus, Web of Science, Embase, and EBSCO. A thorough search of gray literature was also carried out via the following databases: Word Wide Science, the Brazilian Digital Library of Theses and Dissertations (BDTD), and Google Scholar.

The references of the selected articles were checked to identify new studies not found in the previous searches, in accordance with the previously established inclusion criteria. Taking the inclusion criteria into account, the PubMed search strategy was developed using Medical Subject Headings (MeSH), as shown in Table 1. This strategy was adapted according to the specificities of each database used.

**Table 1:**
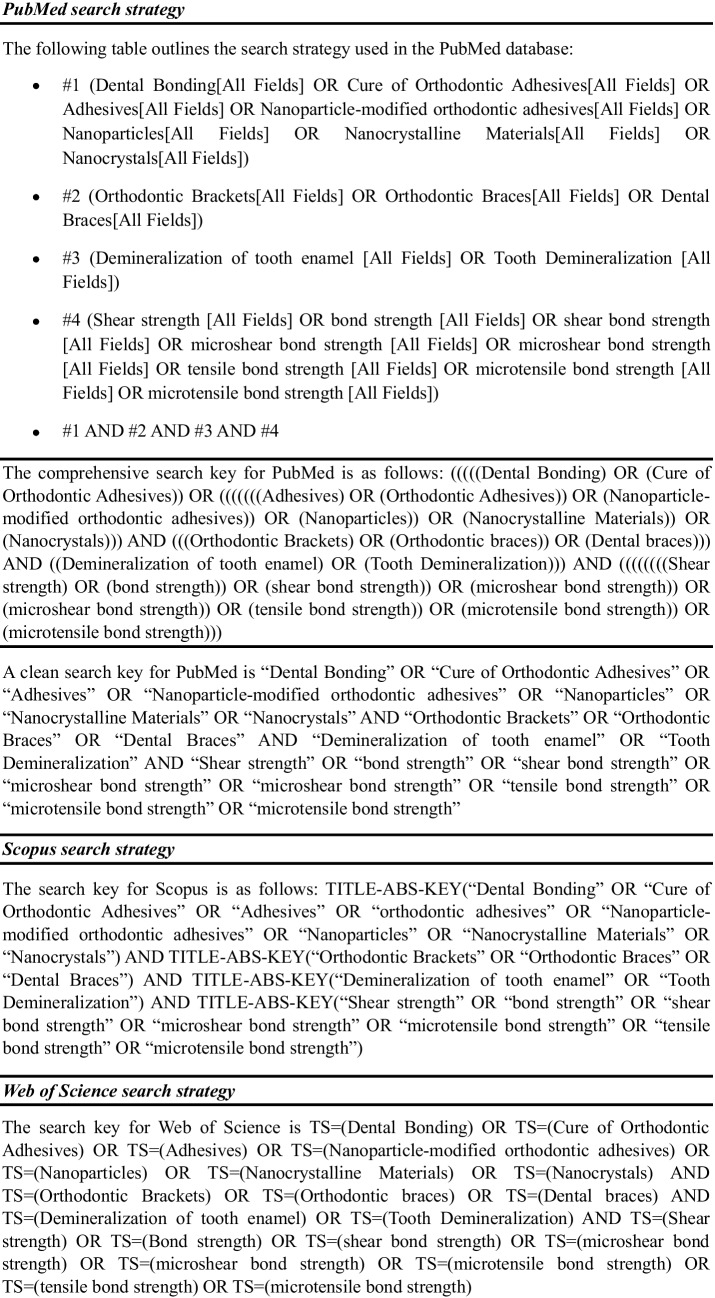

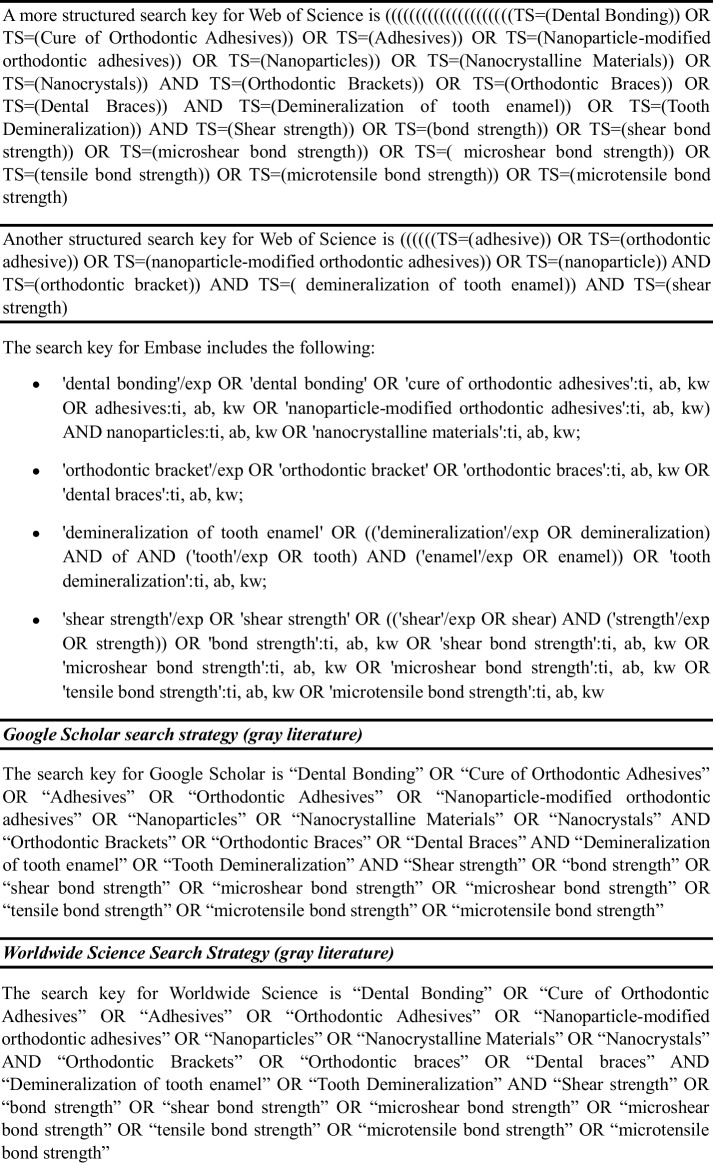
Search strategy

The final search results were exported to Rayyan (https://www.rayyan.ai/), and duplicates were removed.

### Selection of evidence sources

Two reviewers independently screened the studies and selected them on the basis of their titles and abstracts. The preselected articles were subsequently read in full, identifying the articles that were relevant to the research and whether the inclusion criteria were met. Disagreements between the reviewers were resolved by discussion and in collaboration with a third reviewer to achieve consensus among all reviewers.

### Data collection process and synthesis of results

The essential elements found in each publication were extracted and synthesized by two independent reviewers via a structured data tabulation tool designed for this study.

The data extracted included details on authorship, year of publication, type (article, dissertation and government documents), nanoparticle studied, description of the experimental and control groups, and the main findings relevant to the aim of this review.

### Research setting

This scoping review was conducted at the Federal University of Paraíba (UFPB), located in João Pessoa, Brazil. The study is part of the research activities developed within the Graduate Program in Dentistry at UFPB, which supports investigations focused on innovative materials and technologies in oral health. This review aligns with the program’s research on advanced biomaterials and translational applications in orthodontics.

## Results

### Search results

**Fig. 1:**
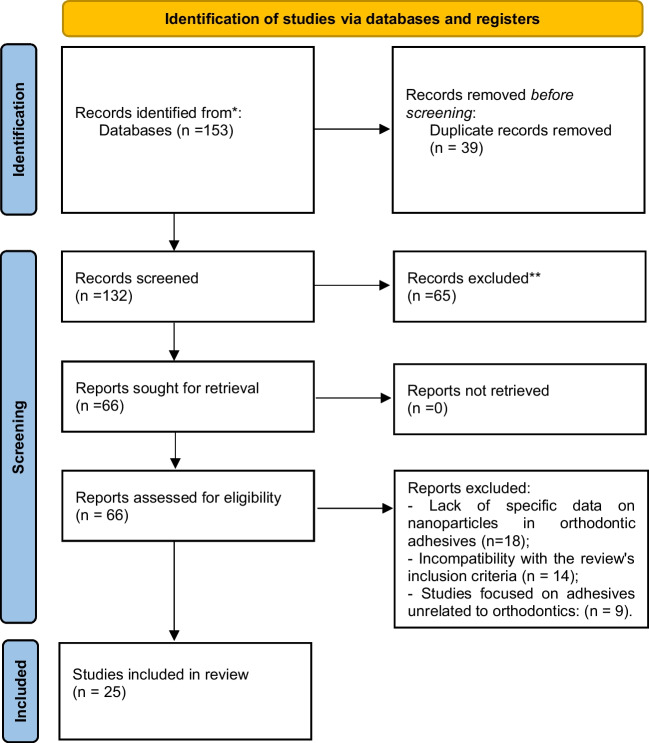
Prisma 2020 flow diagram for new systematic reviews that included searches of databases and registers only

The study selection process began with a comprehensive search across multiple databases, yielding 153 articles. After 39 duplicates were removed during the initial screening, 132 articles were subjected to title and abstract evaluation. At this stage, 65 articles were excluded for failing to meet the inclusion criteria. Among the 66 articles reviewed in full, 41 were excluded for various reasons. Some studies lacked sufficient data on the application of nanoparticles in orthodontic adhesives, focusing only superficially on their incorporation without detailing specific effects. Others presented significant methodological issues, such as the absence of control groups, incomplete experimental descriptions, or the use of nonreproducible testing protocols. Additionally, a subset of articles focused on adhesives used in other dental specialties, such as restorative dentistry or prosthodontics, which fell outside the scope of this review. Ultimately, 25 studies fulfilled all the inclusion criteria and were included in the final analysis (Fig. 1).

### Study characteristics

The 25 studies included in this scoping review, published between 2007 and 2024, exhibit a wide range of experimental designs and methodologies (Table 2). The majority (72%) employed in vitro models to assess the antimicrobial, mechanical, and biocompatibility properties of nanoparticle-modified orthodontic adhesives. Only 16% utilized in vitro methodologies, which more accurately replicate clinical conditions. Preclinical studies are scarce (12%), highlighting the need for research aimed at translating laboratory findings into clinical applications.

**Table 2:** Characteristics of the included studies

Author/Year	Country	Type of study	Nanoparticle	Adhesive experimental	Adhesive Control	Mechanical test	Antimicrobial activity/Desmineralization
Ahmed, et al. 2022^[6]^	Pakistan	In vitro	Silver nitrate (AgNPs) coated with Augmentin	Transbond XT modified with coated nitrate at 2.5% (G1); 5% (G2); 10% by weight (G3)	Transbond XT	Shear bond streng	*S. aureus e E. coli*
Mohhamadi, et al. (Mohammad et al., 2022)^[4]^	Saudi Arabia	Systematic review and meta-analysis	Silver nitrate (AgNPs)	Experimental adhesives with AgNPs	Adhesives without AgNPs	Very heterogeneous studies; 4 studies evaluated through shear testing	*Streptococcus mutans* as the main species (n = 10), followed by *Lactobacillus acidophilus* (n = 3), *Streptococcus sanguinis* (n = 2), and *Lactobacillus sorbius* (n = 1)
Jiali na, et al. 2024	China	In vitro	Mesoporous silica loaded with arginine (Arg@MsNs)	Transbond XT modified with Arg@MSNs at 0.5% (G1), 1% (G2), and 2% by weight (G3)	Transbond XT	Shear bond streng with thermomechanical aging	*S. mutans*
ChaichAna, et al. 2022	Thailand	In vitro	Monohydrated calcium phosphate/Sr-bioactive glass nanoparticles (Sr/CaP) and andrographolide	Five experimental formulations with varying concentrations of Sr-BGNPs/MCPM (Sr/Ca) and andrographolide: 5% (G1) and 10% by weight (G2)	Transbond XT	Shear bond streng with thermomechanical aging	*S. mutans*
Choi, et al. 2021	Korea	In vitro	Mesoporous Bioactive Glass (MBN)	Ortho Connect Flow (SAR) with mesoporous bioactive glass nanoparticles (MBN) at 0% (G1), 1% (G2), 3% (G3), and 5% by weight (G4)	Transbond XT	Shear bond streng	pH cycling was used to test the effects of demineralization and remineralization *S. mutans; P. gingivalis*
Dunn 2007	United States of America	In vitro	Amorphous Calcium Phosphate (ACP)	Aegis Ortho containing amorphous calcium phosphate	Transbond XT	Shear bond streng	No microbiological and demineralization tests were performed
FarzAnegan, et al. 2021	Iran	In vitro	Chitosan and TiO_2_	Transbond with 0.5% (G1), 1% (G2), and 1.5% (G3) of each (chitosan and TiO_2_)	Transbond XT	Shear bond streng with thermomechanical aging	No microbiological and demineralization tests were performed.
Kamran, et al.2021	Saudi Arabia	In vitro	Nanoparticles of Nanoplatina (NSPs) loaded with Poly-L-Lactic Acid (PLGA)	Transbond with NSPs with PLGA at 2.5% (G1) and 5% by weight (G2)	Transbond XT	Shear bond streng with thermomechanical aging	*S. mutans*
Kotta, et al. 2020	Índia	In vitro	TiO_2_	Adhesive with 1% TiO_2_	Conventional adhesive (brand not named)	Shear bond streng	*Streptococcus mutans and Lactobacillus acidophilus*
Ma et al. 2017	United States of America	In vitro	Amorphous Calcium Phosphate (ACP)	GC Ortho with amorphous calcium phosphate	Transbond XT (TB) (G1); GC Ortho (G2)	Shear bond streng	Demineralization tested with altered pH cycles
Melo, et al. 2017	United States of America	In vitro	Amorphous Calcium Phosphate (ACP)	Transbond containing NACP at 10% (G1), 15% (G2), 20% (G3), 30% by weight (G4)	Transbond XT	Shear bond streng	pH cycling
Mirhashemi, et al. 2023	Iran	In vitro	Emodin (ENPs)	BracePaste with ENPs at 0.5% (G1), 1% (G2), and 2% by weight (G3)	BracePaste	Shear bond streng with thermomechanical aging	*Streptococcus mutans, Lactobacillus acidophilus e Candida albicans*
Mollabashi, et al. (Mollabashi et al., 2023)	Iran	Randomized clinical trial Split mouth	TiO_2_	Transbond with 1% TiO_2_	Transbond XT	Did not perform mechanical tests	*S. mutans*
Hyung-Jin Nam et al. 2019	Korea	In vitro	Bioactive Glass with Fluorine (F)	Bioactive glass with 2.5% F + Transbond XT Low Flow 1.3% (G1) and 5% (G2)	Transbond XT	Vickers hardness test; Shear bond streng	*S. mutans* pH cycling
Se Young Park et al. 2020	Korea	In vitro	Methacryloyloxyethyl Phosphorylcholine (MPC) + Mesoporous Bioactive Glass (MBN)	Methacryloyloxyethyl Phosphorylcholine (MPC) + Mesoporous Bioactive Glass (MBN) + CharmFil Flow (3% MPC, 5% MPC, 3% MPC + 3% MBN, and 3% MPC + 5% MBN) e 3% MPC + 5% MBN)	CharmFil Flow	Shear bond streng	*S. mutans; E. coli*
Maryam, P. et al. 2012	Iran	In vitro	TiO_2_	Transbond XT with 1% TiO_2_	Transbond XT	Shear bond streng	*S. mutans*
Rahmanpanah, S. et al. (Rahmanpanah et al., 2023)	Iran	In vitro	n-Hydroxyapatite (HA)	Experimental composites containing 2% (HA2) (G1); 5% (HA5) (G2); and 10% (HA10) (G3) (G1); 5% (HA5) (G2); e 10% (HA10) (G3)	Transbond XT	Shear bond streng; microhardness	pH cycling
Nozha M. Sawan et al. (Nozha et al., 2022)	Saudi Arabia	In vitro	Graphene + Ag	Transbond XT + AG-GS (0.35%) (G1); AG-GS (0.55%) (G2)	Transbond XT	Shear bond streng with thermomechanical aging	*L. acidophilus*
Seifi et al. 2024	Iran	In vitro	Bioactive Glass + Ag (nBG@A)	GC Ortho Connect orthodontic composite containing 1% (G1), 3% (G2), and 5% (G3) of nBG@A	GC Ortho Connect orthodontic composite	Shear bond streng	*S. mutans*
D Tivanani, et al. 2024	India	Systematic review and meta-analysis	TiO_2_	Experimental adhesives with TiO_2_	Experimental adhesives without TiO_2_	Only seven out of the 10 included studies evaluated shear bond strength	Antibacterial activity was assessed in five out of the 10 included studies (various bacteria)
Uehara, LM et al. (Uehara et al., 2024)	Brazil	In vitro	Vanadate decorated with silver nanoparticles (βAgVO_3_)	Transbond XT + 2.5% (βAgVO_3_) (G1); 5% (βAgVO3) (G2)	Transbond XT	Shear bond streng with thermomechanical aging; microhardness	*S. mutans e S. sanguinis*
Xian-Ju, X. et al. 2016	China	In vitro	Amorphous Calcium Phosphate (ACP)	Experimental composite + ACP 40%	Transbond XT	Shear bond streng	Did not perform microbiological and demineralization tests
Jianru, Yi et al. 2019	China	In vitro	Calcium Fluoride- nCAF_2_	Experimental glass ionomer composite with resin + nCaF2 40% and 2% and 3% by weight of DMAHDM	Transbond -XT; Ortho GC	Shear bond streng	*S. mutans* Colony-forming units (biofilm)
Qihan Y et al. 2022	China	In vitro	Polycaprolactone–gelatin–silver nanoparticles (PCL–gelatin–AgNPs)	Transbond XT + PCL–gelatin–AgNPs: 1% (G1); 5% (G2); 10% (G3); and 15% by weight (G4)	Transbond XT	Tensile strength	*S. mutans*
Ling Zhang et al. 2016	China	In vitro	Amorphous Calcium Phosphate (ACP)	Experimental composite + CaP (40%)CaP(40%)	Transbond XT; Vitreme	Shear bond streng with thermomechanical aging	Did not perform microbiological and demineralization tests

Geographically, the studies were predominantly conducted in the United States, China, and Iran, reflecting that these countries play leading roles in advancing research on nanoparticles in orthodontics. For example, Iran contributed significantly to the field, as evidenced by studies such as those by Farzanegan et al. (2021), Rahmanpanah et al. (2023), and Mirhashemi et al. (2023), revealing the region's growing investment in innovative dental research.

Among the various nanoparticles studied, AgNPs stand out as the most extensively investigated, featuring 56% of the studies. Ahmed et al. (2022) and Jiali An et al. (2024) demonstrated the potent antimicrobial effects of AgNPs, particularly against *Streptococcus mutans* and *Escherichia coli*. Additionally, titanium oxide (TiO_2_) nanoparticles have been highlighted in studies such as those by Kotta et al. (2020) and Maryam et al. (2013), with a focus on their ability to increase adhesive strength and reduce bacterial adhesion. Hydroxyapatite (HA), which was examined by Rahmanpanah et al. (2023), has shown potential for improving mechanical properties while promoting enamel remineralization.

Innovative materials such as mesoporous silica loaded with arginine (Arg@MsNs) and β-vanadate silver phosphate (βAgVO_3_) were also explored. For example, Jiali An et al. (2024) highlighted the benefits of Arg@MsNs in maintaining mechanical performance under thermomechanical stress, whereas Uehara et al. (2024) demonstrated the dual advantages of βAgVO_3_ in enhancing bond strength and inhibiting microbial growth.

Despite these promising findings, significant gaps remain in the clinical translation of these materials. The current literature underscores the necessity for long-term studies that address safety, durability, and real-world performance. Future research should prioritize robust clinical trials to establish the efficacy and safety of these nanoparticle-modified adhesives, ultimately bridging the gap between experimental innovation and practical orthodontic applications.

### Synthesized results

The studies reviewed offer a comprehensive perspective on the properties of nanoparticle-modified orthodontic adhesives, revealing substantial progress in antimicrobial performance, biocompatibility, and adhesive strength.

### Biocompatibility

The biocompatibility of orthodontic adhesives modified with nanoparticles is generally favorable, provided that the nanoparticle concentrations are carefully controlled. For example, Rahmanpanah et al. (2023) evaluated adhesives modified with nanohydroxyapatite at concentrations of 2%, 5%, and 10% by weight and reported minimal cytotoxic effects and favorable biocompatibility profiles. Similarly, Mirhashemi et al. (2023) reported that adhesives containing emodin nanoparticles (ENPs) at 0.5%, 1%, and 2% by weight exhibited low cytotoxicity to epithelial cells and fibroblasts. Farzanegan et al. (2021) also demonstrated excellent biocompatibility with adhesives incorporating chitosan and TiO₂ nanoparticles at concentrations of 0.5%, 1%, and 1.5% by weight, promoting enhanced cell proliferation and differentiation.

In contrast, higher concentrations of nanoparticles have been associated with potential cytotoxic effects. Although Mohhamadi et al. (2022) conducted a systematic review suggesting that increased concentrations of AgNPs could negatively impact cellular viability, specific experimental data on cytotoxicity rates were not directly provided. These findings highlight the critical need for precise dosing to balance antimicrobial efficacy and biological safety. Similarly, although Kotta et al. (2020) evaluated adhesives containing 1% TiO₂, their study focused on their mechanical properties and antibacterial activity without specific cytotoxicity assessments. However, other studies have indicated that excessive TiO₂ concentrations may disrupt cellular homeostasis, reinforcing the importance of controlled incorporation levels.

The biocompatibility of orthodontic adhesives also varies according to the type of nanoparticle incorporated. Although studies such as Rahmanpanah et al. (2023) demonstrated favorable biological responses with HA-NPs at concentrations of 2%, 5%, and 10% by weight, data on direct comparisons between different nanoparticle types, such as ZnO-NPs and AgNPs, remain limited. With respect to mesoporous silica nanoparticles loaded with arginine, Jiali An et al. (2024) reported adhesives with preserved mechanical properties, suggesting the potential for biocompatibility, although direct assessments of cytotoxicity were not performed. These findings indicate that both the nanoparticle type and its concentration are crucial factors to be optimized in the development of orthodontic adhesives.

Adhesives incorporating innovative nanoparticles such as βAgVO₃ and Sr/CaP formulations have shown encouraging results regarding material performance and antimicrobial activity. Uehara et al. (2024) reported that adhesives containing βAgVO₃ nanoparticles at 2.5% and 5% maintained favorable mechanical properties and exhibited strong antibacterial effects against *Streptococcus mutans* and Streptococcus sanguinis, although biocompatibility assessments were not directly performed. Similarly, Chaichana et al. (2022) investigated adhesives modified with Sr-bioactive glass nanoparticles combined with monohydrate calcium phosphate (Sr/CaP) at 5% and 10% concentrations, which maintained mechanical integrity and antibacterial potential, suggesting their promising application in orthodontic materials.

These findings highlight the need for further investigations aimed at identifying the optimal types, concentrations, and combinations of nanoparticles. The establishment of standardized biocompatibility testing protocols is essential to ensure consistency and enable reliable cross-study comparisons. These advancements will contribute to the refinement of nanoparticle-modified adhesives, enhancing both their safety and clinical effectiveness.

### Adhesion strength

The incorporation of nanoparticles into orthodontic adhesives generally enhances adhesion strength, particularly by improving resistance to moisture and mechanical stress. Studies involving different types and concentrations of nanoparticles have revealed varying degrees of success. For example, Uehara et al. (2024) demonstrated that adhesives modified with βAgVO₃ nanoparticles maintained or improved shear bond strength after thermomechanical aging, suggesting that these nanoparticles can reinforce adhesive durability under simulated oral conditions. Similarly, adhesives incorporating nanohydroxyapatite (HA-NPs), as reported by Rahmanpanah et al. (2023), exhibited increased mechanical performance, including increased shear bond strength and microhardness, supporting the role of nanoparticles in reinforcing the resin matrix and enhancing resistance to environmental stressors.

In a related study, Farzanegan et al. (2021) investigated the effects of incorporating chitosan and TiO₂ nanoparticles into orthodontic adhesives and reported that these modifications improved shear bond strength, particularly after thermomechanical aging, demonstrating the potential of these nanoparticles to enhance mechanical performance. Similarly, Kotta et al. (2020) reported that adhesives modified with 1% TiO₂ nanoparticles presented increased shear bond strength compared with conventional adhesives, suggesting that TiO₂ incorporation may reinforce the resin matrix and increase its resistance to mechanical and environmental challenges.

AgNPs are among the most frequently studied nanomaterials for orthodontic adhesive modification. Ahmed et al. (2022) incorporated AgNPs coated with Augmentin into Transbond XT at concentrations of 2.5%, 5%, and 10%, reporting that increased nanoparticle concentrations did not yield proportional improvements in shear bond strength, suggesting that excessive nanoparticle loading might interfere with the adhesive matrix. Similarly, Seifi et al. (2024) evaluated orthodontic composites containing bioactive glass nanoparticles doped with silver (nBG@Ag) and reported that while moderate incorporation maintained adhesive strength, higher concentrations could compromise the structural integrity of the resin matrix.

Amorphous calcium phosphate nanoparticles, studied by Ma et al. (2017) and Melo et al. (2017), demonstrated a positive influence on bond strength, particularly when incorporated at moderate concentrations ranging from 10 to 20% by weight. These studies indicated that ACP nanoparticles not only increased shear bond strength but also contributed to enamel remineralization under demineralization challenge conditions, offering dual benefits for adhesion and protection against enamel demineralization. Similarly, Xian-Ju et al. (2016) reported increased shear bond strength with the incorporation of ACP nanoparticles into experimental adhesives, emphasizing the role of the material in improving mechanical performance. These findings support the potential of ACP-modified adhesives to simultaneously reinforce adhesion and promote enamel health.

Hydroxyapatite nanoparticles, as studied by Rahmanpanah et al. (2023), were found to improve the shear bond strength and microhardness of orthodontic adhesives while maintaining favorable biocompatibility. Additionally, Se Young Park et al. (2020) reported that the combination of methacryloyloxyethyl phosphorylcholine (MPC) with mesoporous bioactive glass nanoparticles (MBNs) enhanced both mechanical properties and antimicrobial activity, with the incorporation of MBN leading to increased shear bond strength. These results highlight the synergistic potential of combining different nanoparticles to optimize adhesive performance.

In contrast, Qihan Y et al. (2022) reported that incorporating PCL–gelatin–AgNPs into Transbond XT adhesives resulted in improved tensile strength at lower concentrations (1%–5%), but higher concentrations (10%–15%) did not provide proportional gains, underscoring the importance of optimizing nanoparticle loading to prevent adverse effects on adhesive performance.

These findings collectively emphasize the significant potential of nanoparticles to increase adhesion strength but also underscore the critical importance of optimizing nanoparticle concentrations and combinations. High nanoparticle concentrations might impair adhesive performance; therefore, a precise formulation of nanoparticle-loaded adhesives is essential for achieving the optimal balance between enhanced adhesion and other properties, such as antimicrobial activity and biocompatibility. Future research should focus on refining nanoparticle integration methods and developing standardized protocols for testing the adhesive properties of these modified materials to ensure consistent and reliable results across different studies.

### Interaction of properties

Most studies have focused on evaluating antimicrobial properties, biocompatibility, or adhesion strength separately, with limited exploration of the interactions among these properties. Jiali Na et al. (2024) evaluated orthodontic adhesives modified with mesoporous silica nanoparticles loaded with arginine (Arg@MSNs) and reported that the adhesives maintained adequate shear bond strength after thermomechanical aging, along with improved antimicrobial activity against *Streptococcus mutans*. However, no direct biocompatibility evaluation has been reported. Similarly, Farzanegan et al. (2021) assessed the addition of chitosan and TiO₂) nanoparticles and observed enhanced mechanical properties after thermomechanical aging; nonetheless, no antimicrobial or biocompatibility assessments were performed, highlighting the gap in studies simultaneously analyzing multiple properties.

Additionally, Mirhashemi et al. (2023) evaluated orthodontic adhesives containing emodin nanoparticles (ENPs) and reported that their incorporation improved shear bond strength after thermomechanical aging, along with antimicrobial activity against *Streptococcus mutans*, *Lactobacillus acidophilus*, and *Candida albicans*. However, no biocompatibility assessments have been reported.

Similarly, Uehara et al. (2024) investigated the incorporation of vanadate decorated with silver nanoparticles and reported that adhesives exhibited improved shear bond strength after thermomechanical aging and increased antimicrobial activity against *Streptococcus mutans* and Streptococcus sanguinis. No direct comparison with AgNPs or specific biocompatibility evaluation has been described. This variability among different nanoparticle types underscores the challenge of optimizing all critical properties simultaneously.

Despite these promising results, the limited number of studies addressing the simultaneous optimization of mechanical, antimicrobial, and biocompatibility properties highlights a significant gap in the literature. Kotta et al. (2020) evaluated adhesives containing TiO₂ nanoparticles and reported improved shear bond strength and antimicrobial activity against *Streptococcus mutans* and *Lactobacillus acidophilus* but did not assess their biocompatibility, reinforcing the need for careful formulation control. Moreover, Jianru Yi et al. (2019) investigated adhesives containing calcium fluoride nanoparticles (nCaF₂) and emphasized the importance of incorporating protocols that simulate clinical conditions, such as evaluating mechanical and antimicrobial properties under realistic stress conditions, although cytotoxicity was not assessed. These findings suggest that dynamic and integrated testing approaches are necessary to better predict material performance in oral environments.

The complexity of balancing antimicrobial efficacy, biocompatibility, and adhesion strength calls for a more integrated approach in future research. Advanced testing protocols and modeling techniques should be employed to assess the potential synergistic or antagonistic effects of nanoparticle incorporation.

### Methodological rigor and variability

Most studies included in this review demonstrated commendable methodological rigor, presenting well-defined protocols and detailed reporting of their findings. For example, Farzanegan et al. (2021) provided a clear description of nanoparticle concentrations (chitosan and TiO₂) and performed shear bond strength tests with thermomechanical aging, although they did not evaluate antimicrobial or biocompatibility properties. Similarly, Rahmanpanah et al. (2023) detailed the preparation of adhesives containing n-hydroxyapatite at different concentrations and assessed their mechanical properties, such as shear bond strength and microhardness, under defined test conditions. Uehara et al. (2024) also demonstrated methodological transparency by specifying βAgVO₃ concentrations and performing shear bond strength and microhardness testing after thermomechanical aging. Similarly, Jiali Na et al. (2024) described the incorporation of Arg@MSNs and evaluated the shear bond strength with thermomechanical aging and antimicrobial activity against *S. mutans*. Such methodological detail is essential for enabling future replication and for fostering consistent comparisons across studies.

However, significant variability in experimental protocols emerged as a recurring limitation across the studies. Differences in nanoparticle concentrations, adhesive formulations, and testing conditions—such as variations in thermomechanical aging procedures and microbial assays—pose substantial challenges to direct comparisons. For example, Mirhashemi et al. (2023) and Kotta et al. (2020) both employed in vitro models using ENPs (emodin nanoparticles) and TiO₂ nanoparticles, respectively, but differed in nanoparticle types, concentrations, and specific test conditions. While both evaluated shear bond strength, differences in microbial strains, humidity, temperature control, and the absence of biocompatibility tests complicate efforts to establish standardized benchmarks. Furthermore, the diverse incorporation of materials such as n-hydroxyapatite (HA), mesoporous silica loaded with arginine (Arg@MSNs), and βAgVO₃ across different experimental settings highlights the pressing need for the adoption of uniform methodologies to increase the comparability of results regarding the efficacy and safety of nanoparticle-modified adhesives.

Moreover, while many studies have been well structured and accessible, some, such as Ahmed et al. (2022), presented highly technical language and intricate methodological descriptions that could hinder replication efforts, particularly for researchers who are less familiar with nanoparticle technologies. Although Ahmed et al. (2022) clearly described the incorporation of AgNPs coated with Augmentin into the adhesive and detailed shear bond strength testing and antimicrobial assays, the absence of simplified explanations regarding nanoparticle synthesis, coating processes, and dispersion techniques may create barriers for broader reproducibility.

To address these issues, future studies should prioritize greater uniformity in experimental design and reporting standards. Simplifying and streamlining methodological descriptions, including clear specifications of nanoparticle types, concentrations, preparation methods, and test conditions, would increase reproducibility, especially for researchers entering the field of nanoparticle-enhanced orthodontic adhesives. Furthermore, adopting standardized reporting guidelines—such as CONSORT or PRISMA, adapted for in vitro laboratory studies in dental materials—could foster greater transparency and consistency, facilitating more robust comparisons across studies.

### Limitations of the assessments

Several critical limitations emerged from the studies included in this review, particularly concerning variability in experimental protocols and the constrained scope of the research. A major inconsistency was observed in the methodologies used across studies, with differences in nanoparticle concentrations, adhesive formulations, and testing environments, which made direct comparisons challenging. For example, while Farzanegan et al. (2021) used uniform test conditions to evaluate adhesives modified with chitosan and TiO₂, other studies, such as Zhang et al. (2016), incorporated amorphous calcium phosphate (ACP) into experimental composites and employed different concentrations and testing approaches, including thermomechanical aging, without standardized antimicrobial evaluation. This inconsistency hinders the ability to establish standardized testing parameters and reliable benchmarks for assessing the efficacy and mechanical performance of nanoparticle-modified orthodontic adhesives.

Another major limitation identified in the reviewed studies was the predominance of controlled laboratory (in vitro) settings. While such studies provide important initial insights, they fail to replicate the dynamic and complex conditions of the oral environment. As highlighted by Ling Zhang et al. (2016) and Mirhashemi et al. (2023), the absence of clinical trials severely restricts the real-world applicability of the findings. Key factors such as saliva flow, temperature fluctuations, mechanical forces from mastication, and biofilm maturation are not adequately simulated under in vitro conditions, which may lead to overestimations of adhesive performance in clinical practice. For example, although nanoparticles such as amorphous calcium phosphate (ACP), emodin nanoparticles (ENPs), AgNPs, and TiO₂ have demonstrated promising antimicrobial properties in laboratory models, their effectiveness within the oral cavity—subject to various and multifactorial challenges—remains uncertain.

The duration of most experiments also presented a significant limitation. Many studies have focused on relatively short-term evaluations of antimicrobial and mechanical properties, typically lasting only a few days or weeks. This limited duration restricts the ability to assess the long-term performance, safety, and material stability of nanoparticle-modified adhesives over extended periods. For example, studies such as Rahmanpanah et al. (2023) and Jiali Na et al. (2024) demonstrated promising initial findings, with improvements in mechanical properties and antimicrobial activity, but lacked longitudinal data to evaluate how these materials perform over time, particularly regarding their cytotoxicity, adhesion strength retention, and durability. Given the complexity of the oral environment, which involves daily exposure to masticatory forces, saliva, and pH fluctuations, long-term studies are crucial to determine the real-world effectiveness and safety of these materials.

These limitations highlight the need for more standardized, robust, and clinically relevant research designs. Future studies should prioritize long-term evaluations under simulated clinical conditions that more accurately replicate oral challenges, including factors such as saliva composition, cyclic mechanical loading, and temperature variations.

### Recommendations

To advance the field of nanoparticle-modified orthodontic adhesives, future studies should focus on addressing current gaps and enhancing their clinical applicability. A key priority is the development of robust clinical trials to evaluate the efficacy, safety, cost-effectiveness, and impact of these adhesives on patients'quality of life across diverse populations and over extended periods. Such trials should aim to replicate real-world conditions by incorporating variables such as saliva composition, oral microbiota, temperature fluctuations, and masticatory forces, ensuring that results are applicable beyond controlled laboratory environments. Although clinical studies are scarce, the importance of simulating dynamic oral factors has been emphasized in laboratory research, such as in the work of Ling Zhang et al. (2016), highlighting the critical need for more realistic evaluation protocols to better predict in vivo performance.

Standardization of experimental protocols is also critical. Uniform approaches for determining nanoparticle concentrations, adhesive formulations, and testing methodologies are necessary to facilitate meaningful comparisons and synthesis of findings across studies. Research such as that conducted by Uehara et al. (2024) and Rahmanpanah et al. (2023) emphasized the importance of standardizing test conditions, including thermomechanical aging procedures and mechanical property evaluations such as shear bond strength and microhardness tests, to increase result reliability and reporting consistency across different research groups.

Additionally, expanding research to explore novel nanoparticles is highly recommended. While silver nanoparticles, titanium dioxide, and mesoporous silica-based nanoparticles have been extensively studied, future attention should focus on newer materials with multifunctional properties. NPs derived from bioactive ceramics, carbon-based materials, and hybrid composites are of particular interest because of their potential to simultaneously increase antimicrobial activity, mechanical strength, and biocompatibility. For example, βAgVO₃, investigated by Uehara et al. (2024), demonstrated improvements in both antimicrobial performance and shear bond strength, underscoring the potential of emerging nanoparticle systems to surpass traditional modifications.

Long-term trials are indispensable for assessing the durability and safety of nanoparticle-modified orthodontic adhesives. Techniques such as thermomechanical aging, employed by Uehara et al. (2024), provide valuable preliminary insights into how these materials might perform over extended periods, simulating months or years of clinical function without necessitating excessively prolonged studies. Long-term assessments are crucial for generating robust data on material stability, cytotoxicity, and mechanical resilience, which are essential factors for the clinical adoption and success of these advanced adhesive systems.

## Discussion

The studies included in this scoping review highlight the promising potential of nanoparticles in orthodontic clinical practice while emphasizing the need for rigorous clinical validation of the positive results observed in laboratory settings. The incorporation of nanoparticles into orthodontic adhesives represents an innovative strategy to address traditional challenges associated with fixed appliances, particularly enamel demineralization (Jianru Yi et al. 2019; Hyung-Jin Nam et al. 2019; Farzanegan et al. 2021). This advancement has the potential to significantly improve patient outcomes by mitigating common complications related to biofilm accumulation and enamel damage.

Among the nanoparticles studied, silver nanoparticles have been the most extensively investigated and are consistently associated with potent antimicrobial activity, particularly in the inhibition of bacterial biofilm formation. These properties are crucial for preventing the development of oral diseases in patients undergoing orthodontic treatment. Furthermore, studies indicate that when incorporated at moderate concentrations, AgNPs can maintain an acceptable biocompatibility profile. However, despite these promising findings, further research is necessary to fully elucidate potential adverse effects related to nanoparticle toxicity, especially considering the complex and dynamic nature of the oral environment, which may impact the material's clinical performance (Mohhamadi et al. 2022; Nozha M. Sawan et al. (Nozha et al., 2022)).

Similarly, titanium dioxide nanoparticles have demonstrated antimicrobial properties and satisfactory biocompatibility. However, some studies have indicated a reduction in shear bond strength when TiO₂ is used in adhesive formulations, emphasizing the importance of optimizing nanoparticle concentrations and formulations to avoid detrimental effects on adhesive performance. This variation in outcomes highlights the complexity of nanoparticle incorporation and the need for standardization in experimental protocols. Graphene nanoparticles, both in pure form and in combination with silver, as well as β-AgVO₃, also exhibit strong antimicrobial potential, suggesting their potential for use in future orthodontic adhesives (Poosti et al. 2013; Kotta et al. 2020; Mollabashi et al. 2023; Tivanani et al. 2024; Nozha et al. 2022; Uehara et al. 2024).

Bioactive materials, such as MBN, particularly when combined with silver nanoparticles or specific monomers such as 2-MPC, demonstrated superior enamel remineralization capacity and enhanced biocompatibility. The combination of 2-MPC with these materials not only improved antimicrobial resistance but also minimized cytotoxicity, which is crucial for the long-term safety of orthodontic patients (Park et al. 2020; Choi et al. 2021; Seifi et al. 2024). These developments represent a significant step toward creating adhesives that promote enamel health while simultaneously providing antimicrobial protection.

NPs derived from inorganic compounds, such as ACP, n-hydroxyapatite, and mesoporous silica loaded with arginine, have shown significant potential in preventing enamel demineralization and promoting enamel remineralization. Furthermore, innovative combinations such as Sr/CaP and andrographolide demonstrated robust antimicrobial properties while maintaining or improving the mechanical strength of the adhesives. These materials could lead to adhesives that not only prevent enamel caries but also reinforce the structure of enamel, making them highly beneficial for orthodontic applications (Dunn 2007; Zhang et al. 2016; Xie et al. 2017; Ma et al. 2017; Melo et al. 2017; Rahmanpanah et al. 2023; Chaichana et al. 2022; Yuan et al. 2022).

Moreover, natural nanoparticles, such as emodin (ENPs), have emerged as promising alternatives, particularly when used in conjunction with complementary strategies such as antimicrobial photodynamic therapy. These nanoparticles offer a natural approach to antimicrobial protection, but further research is needed to identify the ideal concentrations that maximize antimicrobial efficacy without compromising the shear bond strength of the adhesive (Mirhashemi et al. 2024).

Despite these significant advancements, methodological heterogeneity remains a notable challenge. The lack of standardization in nanoparticle concentrations, types, and experimental protocols complicates direct comparisons of results across studies. Additionally, many studies have been confined to controlled laboratory conditions, which do not accurately replicate the complexity of the dynamic oral environment. Saliva, temperature fluctuations, and mastication forces are critical factors that influence the real-world performance of these adhesives; however, they are often not adequately simulated in laboratory experiments (Arksey & O'Malley 2005; Dunn 2007; Poosti et al. 2013; The JoAnna Briggs Institute, (2014); Zhang et al. 2016; Ma et al. 2017; Melo et al. 2017; Xie et al. 2017; Yi et al. 2019; Nam et al. 2019; Park et al. 2020; Kotta et al. 2020; Pollock et al. 2022; Chaichana et al. 2022; Nozha et al. 2022; Yuan et al. 2022; Mohammad et al. 2022; Muhammad et al. 2022; Ahmed et al. 2022; Farzanegan et al. 2021; Rahmanpanah et al. 2023; Poirier 2023; Mollabashi et al. 2023; Seifi et al. 2024; Tivanani et al. 2024; Uehara et al. 2024; An et al. 2024; Mirhashemi et al. 2023).

The clinical applicability of these formulations requires rigorous clinical trials that assess not only antimicrobial efficacy and mechanical strength but also the long-term safety of nanoparticle-modified adhesives, particularly regarding their potential adverse effects on oral tissues. Therefore, while the presented advancements mark a significant milestone in the development of orthodontic materials, the translation of these findings into clinical practice must be supported by well-designed studies to ensure the viability and effectiveness of these adhesives in real-world settings (Nam et al. 2019; Yi et al. 2019; Farzanegan et al. 2021; Kotta et al. 2020; Choi et al. 2021; Seifi et al. 2024; Mirhashemi et al. 2024).

In clinical practice, nanoparticle-modified orthodontic adhesives may offer distinct advantages for specific patient populations. For example, individuals at high risk of caries—such as those with poor oral hygiene, frequent sugar exposure, or fixed appliances—may benefit from adhesives containing silver (Ahmed et al. 2022), titanium dioxide (Kotta et al. 2020), or mesoporous bioactive glass nanoparticles (Choi et al. 2021; Park et al. 2020; Seifi et al. 2024; Rahmanpanah et al. 2023), which offer increased antimicrobial activity and enamel remineralization. Additionally, patients with periodontal vulnerability may require materials with improved biocompatibility and anti-inflammatory properties. In such cases, adhesives formulated with emodin nanoparticles (Mirhashemi et al. 2024) or bioactive glass associated with methacryloyloxyethyl phosphorylcholine (MPC) (Park et al. 2020; Choi et al. 2021) may provide safer alternatives. These population-specific strategies may contribute to more individualized and effective orthodontic care.

## Conclusion

The incorporation of nanoparticles into orthodontic adhesives has led to promising improvements in adhesion quality, particularly in terms of resistance to moisture and mechanical stress. Nanoparticles such as silver, titanium dioxide, and graphene increase the bond strength of adhesives, which is crucial for the long-term stability of orthodontic appliances. These advancements could lead to stronger and more durable adhesive bonds, reducing the risk of debonding and enhancing overall treatment effectiveness.

However, the clinical application of these adhesives is still limited by variability in nanoparticle concentrations and formulations. Studies have shown that while moderate concentrations of nanoparticles improve the adhesion strength, higher concentrations can disrupt the adhesive matrix, potentially reducing the bond strength. Standardized formulations and further clinical evaluations are needed to ensure consistent performance under real-world conditions.

Thus, while nanoparticle-modified adhesives offer clinical potential for improving adhesion strength, additional clinical trials and refined formulations are necessary to confirm their reliability and feasibility and optimize their use in routine orthodontic practice.

## Data Availability

The authors confirm that the data supporting the findings  of this study are available within the article and its supplementary materials.
